# The Management of Toe Walking in Children with Autism Spectrum Disorder: *“Cast and Go”*

**DOI:** 10.3390/children9101477

**Published:** 2022-09-27

**Authors:** Francesco Manfredi, Flavia Riefoli, Michele Coviello, Daniela Dibello

**Affiliations:** 1 Orthopedic and Trauma Unit, Department of Neuroscience, Pediatric Hospital “Giovanni XXIII”, 70126 Bari, Italy; 2 Orthopaedic and Trauma Unit, Department of Basic Medical Sciences, Neurscience and Sense Organs, School of Medicine, AOU Consorziale Policlinico, University of Bari “Aldo Moro”, Piazza Giulio Cesare 11, 70124 Bari, Italy

**Keywords:** autism, toe walking, serial casting, botulinum toxin

## Abstract

Background: Toe walking is associated with autism spectrum disorders (ASD). Correction of this “behavior” is a health challenge. The toe walker is affected by the contact refusal with the outside world: touching the ground as little as possible, trying to avoid any contact. A structured equines foot is a possible consequence. Method: We present the “Cast and Go” protocol, used in 22 idiopathic toe walker children with ASD treated from 2015 to 2020. The treatment was performed by a single senior experienced doctor with botulinum injection, ankle casts and rehabilitative therapies. All patients underwent pre- and post-treatment clinical evaluation using ankle dorsiflexion angle and casting number as treatment. We aimed to identify the intervention with the shortest acquisition time for the management of toe walking. Results: Our findings demonstrated the baseline ankle dorsiflexion angle influenced the casting number (*p* < 0.01) and male patients had a higher baseline ankle dorsiflexion angle than female patients (*p* < 0.01). No adverse events were observed. Conclusions: These findings suggested that the “Cast and Go” protocol could be a promising, dynamic and effective practice for toe walking disease in ASD patients.

## 1. Introduction

Autism spectrum disorder (ASD) is a complex neurodevelopmental disease that involves communication, social interaction and language in different ways [1]. It manifests itself by repetitive and stereotyped behaviors in different clinical features [2]. The Diagnostic and Statistical Manual of Mental Disorders, 5th ed., eliminated previous subgroups that included several separate conditions: autistic disorder, Asperger syndrome, childhood disintegrative disorder and pervasive developmental disorder not otherwise specified [3].

ASD symptoms can be described in two groups: (1) lack of communication and social interaction or (2) presence of restricted and/or repetitive activities, behaviors and interests. The clinical presentation can be different, associated with psychiatric and medical conditions. Moreover, the symptoms must be manifest in the early developmental period, but not absolutely, and they must compromise social, occupational or other important areas of current functioning. These characteristics are not better explained by intellectual disability or global developmental delay [4,5].

The ASD child’s proprioceptive perception is often so altered as it involves postural and biomechanical adaptations. The psychological attitude towards the external world, as lack of expressiveness and looking up or down, is reflected in the whole body. Moreover, sensorimotor, visual and auditory system interactions may be important contributing factors in postural control in ASD children [6].

The ASD toe walker is affected by outside world contact refusal: touching the ground as little as possible, trying to avoid any contact. This psychological behavior often causes them to walk on tiptoes without a specific medical reason [7]. ASD diagnosis does not involve toe walking, but it could be considered as a “specifier” in the comorbidities section, as described in the DSM-5 [4,5]. Proprioception and sensory changes in ASD are still debated. Hirai M. et al. [8] described ASD tending to use egocentric representations to learn reaching movements towards a target rather than allocentric ones.

Toe walking is considered a normal stage in gait development but should resolve by 3–7 years of age.

The pathological toe walking, on the other hand, is often associated with several diseases. It is a common presentation to the pediatrician, neurologist, podiatrist, and pediatric orthopedic surgeon, with toe walking present at approximately 1% of visits to pediatric orthopedics. The family history is positive between 10 and 85%, and in some cases, an autosomal dominant inheritance is recognized with incomplete expression observed in some families. Different authors proposed two main groups: (1) neurologic/neuromuscular diseases, such as cerebral palsy, muscular dystrophy, Charcot–Marie–Tooth disease, spinal cord or brain injury, tethered cord, spina bifida, schizophrenia; (2) others, such as idiopathic, ASD, limb length discrepancy, clubfoot, tendon or joint contracture, ankylosing spondylitis, unilateral hip dislocation. The pathological walking caused by myotendinous retraction of the sural muscles bilaterally, succeeding in transforming an equine “attitude” into structured clubfoot walking [9]. Many therapy options have been described: no treatment, physical therapy or casting to stretch the gastrocnemius, soleus, and Achilles’ tendon, surgical lengthening of the gastrocnemius, soleus and Achilles tendon, blocking plantar flexion as with an ankle-foot-orthosis or some combination of these. A consensus has not successfully been reached [10].

Particularly important but equally difficult is to recognize idiopathic toe walking (ITW). It is an exclusionary diagnosis supposed when a child toe walks in the absence of any known medical conditions. The prevalence, diagnosis and treatment has been documented, debated and continues to feature in the current literature. There is no medical reason for this gait pattern [11]. Especially useful, the “Toe Walking Tool” [12] questionnaire was designed to prompt the user to identify the indicators or risk factors associated with medical conditions. The tool presented exclusionary questions in a successive order. The aim of questionnaire is to prompt the clinician to refer for further medical investigation. The tool is broken into sections: (1) Demographics, (2) Indicators of trauma, (3) Indictors of neuromuscular influence and (4) Indicators of neurogenic influence. Thanks to this important tool we were able to assess inclusion/exclusion criteria for the study.

Shetreat-Klein et al. [13] showed that about 68% of the ASD children complained a walking disorder. They demonstrated that ASD children had significantly greater joint mobility, more gait abnormalities, and on average, walked 1.6 months later than their non-autistic peers.

Re-education is fundamental for these patients to avoid structured equines. The tendinous contribution of the gastrocnemius and the soleus muscles merge to form the Achilles tendon. Both tendons converge approximately 15 cm proximal to the insertion site [14]. An equinus contracture is characterized by a limitation of at least 10 degrees of passive ankle dorsiflexion with the knee extended and the ankle in neutral position [15]. Less than 10 degrees of dorsiflexion can lead to various compensations in the lower extremity [16,17]. In the literature, 50–70% of children with toe walking measured equinus contracture [18,19].

Possible treatment strategies are described in the literature that include observation, physical therapy, serial casting, orthosis [20] or Achilles’ tendon (heel cord) lengthening surgery [21].

Surgery is often unsuccessful in ASD, as the corrected foot can become equine again due to the child’s behavior recurrence. However, important systematic reviews of the literature [22,23] described that there are not significant differences between the surgery group and conservative treatment in terms of toe walking persistence and after-treatment complications.

These are the reasons why our work investigates less invasive methods associated with prevention with adequate cognitive–behavioral therapy.

The “Cast and Go” protocol provides the combined used of botulinum toxin (one injection alone), serial casting and orthoses associated with physiotherapy to achieve ankle neutral position.

The current study aims to assess the clinical effectiveness of our protocol in ASD patients affected by ITW at long-term follow-up with ankle angle restore and non-pathological walking [24].

## 2. Materials and Methods

### 2.1. Patients and Data Collection

This was a retrospective case study of all patients diagnosed with ASD as the Diagnostic and Statistical Manual of Mental Disorders, 5th edition (DSM-5) criteria.

From January 2015 to December 2020, 40 children affected by ASD were evaluated at the Pediatric Neuro-Orthopedic Surgery of the Orthopedics and Traumatology Unit of the Giovanni XXIII Pediatric Hospital in Bari. This study was approved by the Local Ethical Committee (DG.n° 1563, n. 7086 Prot. n. 0000493).

Inclusion criteria were: (1) autism spectrum disorders (ASD) using DSM-5 criteria [25], (2) ITW using “Toe Walking Tool” [26], (3) gait disorder more than 50% of the time, (4) compliance with our treatment protocol, (5) had not been previously treated with any orthopedic therapeutic protocol, and (6) the family agreed to participate in our study and the patient’s parents/guardian provided written informed consent.

Exclusion criteria were: (1) chronic medical condition such as visual-vestibular problems, (2) associated orthopedic conditions, (3) underlying genetic disorders, (4) global developmental delay, (5) patient’s parents’/guardians’ refusal to provide informed consent.

Each patient was visited by a hospital geneticist who ruled out genetic abnormalities, if necessary, using genetic testing.

Of 40 patients, only 30 children met all the above mentioned criteria. Three patients were noncompliant with the treatment protocol, two patients had underlying genetic disorders, and three patients had associated global developmental delay.

We selected, in the end, 22 children, aged from 4 to 15, with 12 boys and 10 girls, sorted by three subgroups (8 children aged from 4 to 7 years, 8 children aged from 8 to 11 years and 6 teenagers aged from 12 to 15 years).

The following data were collected for all patients: age at presentation, gender, subgroup, baseline and follow-up ankle-dorsiflexion angles (°), casting number as treatment and recurrence after first treatment. Specific data on the socioeconomic status and educational attainment levels of the patients’ families were not recorded. According to the American Academy of Neurology and Child Neurology Society [27], the Gilliam autism rating scale (GARS)—third edition [28] was also included to demonstrate possible clinical variations protocol-dependent on ASD disease applied by the Hospital Psychology Department. The scale consists of 58 items grouped into Restricted/Repetitive Behaviors (13 items), Social Interaction (14 items), Social Communication (9 items) and Emotional Responses (8 items). Total scores were compared before and after protocol treatment. Follow-up was from 1 to 3 years.

We considered ankle degrees as a unique movement arch. An ankle neutral position is measured as 90°. A toe walker, consequently, walks at an angle greater than 90°.

Demographic and main data of the study are reported in Table 1.

### 2.2. Treatment

The “Cast and Go” protocol, used at the Pediatric Unit of Orthopedics and Traumatology of the Giovanni XXIII Pediatric Hospital in Bari for 20 years, was used for all patients. The main features are the following: (a) patient and family compliance as teamwork, (b) the serial casting, (c) botulinum toxin, (d) orthoses, braces and physical and psychological support. After plaster removal, orthoses are used only nightly.

The protocol is divided into 5 steps: (1) careful evaluation of the child and his walking way, (2) bilaterally performed botulinum toxin injection of the sural muscles, (3) casting gradual application 4–7 days after the injection, (4) multidisciplinary approach (physiotherapists, occupational therapists, psychologists and orthopedics) to support the child, during plaster walking, (5) intensive educational treatment performed with braces during the night with ankle joint at least 90 degrees after cast removal.

A 90° of ankle dorsiflexion is the reached goal. If first plaster reaches degrees, the treatment will take 30 days. If not, it will be necessary to perform a new cast correction every 14 days depending on gravity. The maximum overall time of 2–3 consecutive casts never exceeded 35–40 days.

Each patient underwent twice-a-week sessions with an experienced therapist in ASD until the end of protocol, for example, by applying occupational therapy. The whole process was supervised by the Hospital Psychology Department, which provides periodic sessions for the family and for the child. The protocol could be repeated after one year if the ankle-dorsiflexion angle remains more than 90°. The protocol cannot be repeated more than 3 times.

We used a “Toe Walking Tool” to diagnose ITW, excluding other medical disease.

### 2.3. Statistical Analysis

All data were collected and electronically analyzed using SPSS 25.0 (IBM, Armonk, NY, USA). In order to account for non-normality distribution (test Kolmogorov–Smirnov), non-parametric tests were used. Statistical significance “alpha” was fixed to 0.05. Descriptive statistics (mean, standard deviation and percentage) were calculated. Kendall’s tau b test was used to demonstrate correlations between variables, while Mann–Whitney U-test and Wilcoxon signed ranks test were employed to compare variables between different groups. Results are reported in tables.

### 2.4. Outcomes

**Figure 1:**
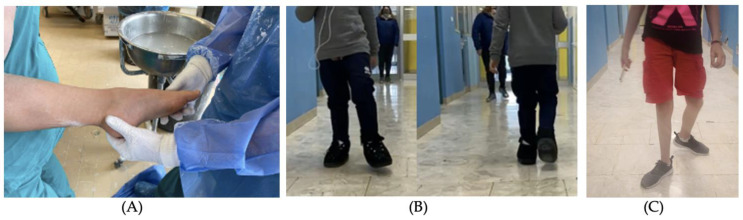
(**A**) Preintervention representation of the participant. (**B**) Participant at 1 week after casting follow-up. (**C**) Participant at 2-year follow-up.

The primary endpoint examined was the correction of the ankle dorsiflexion angle (°) using degrees measured with a medical protractor. The secondary endpoint was failure rate evaluated with recurrence number as the number of patients who repeated the protocol a second time and casting number as treatment. We considered the end of the protocol when the desired angle was reached and recurrence number (Figure 1).

## 3. Results

Twenty-two ASD children, aged from 4 to 15 years, with 12 males and 10 females, were included in this case study. The maximum follow-up was 50 months, with a mean of 37 ± 13 months.

### 3.1. Gender Analysis

We reported a prevalence of males over females (Table 2) and their starting dorsi-flexion angle is statistically significantly worse.

### 3.2. Subgroup Analysis

The baseline ankle dorsiflexion was different for each group. The worst mean value was reported for Group 2.

No statistically significant difference between groups (Table 3) was detected in the baseline ankle-dorsiflexion angle, casting number and recurrence rate.

**Figure 2:**
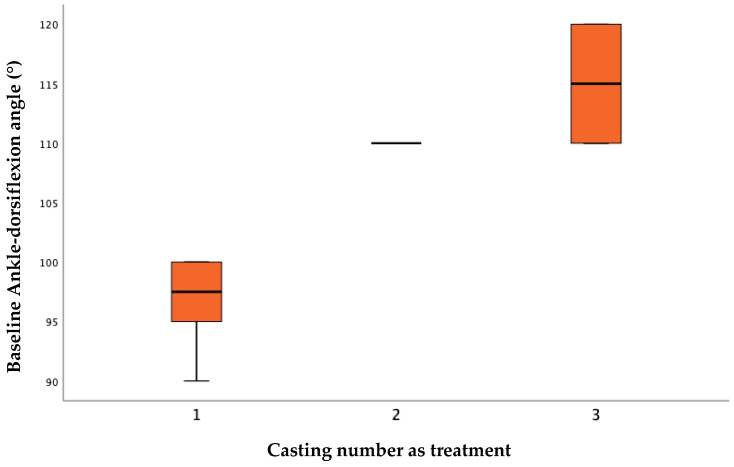
Casting number as treatment and baseline ankle-dorsiflexion angle (°), *p* = 0.001.

Baseline angle statistically influenced treatment, with the larger the angle indicating that more plasters were needed to obtain the correction (Table 4) (Figure 2).

All the ASD patients (100% of the sample) completed the protocol achieving neutral ankle position.

No statistically significant difference after protocol treatment was detected in the GARS-3 scores usign Wilcoxon signed ranks test (maean and standard deviation pre- and post-treatment, 48.11 ± 14.35 versus 49.11 ± 13.03, respectevely, with *p* = 0.66).

## 4. Discussion

The ASD child is a real challenge for the doctor. He has different symptomatologic expressions, not only from the behavioral but also from the postural and psychomotor point of view. According to the DSM-V, the Social Communication and Interaction are really important features. Indeed, Barsotti J. et al. [29] identified specific receptive grammatical deficits that could be or not related to expressive deficits.

Toe walking is a bilateral gait abnormality in which a normal heel strike is absent and most weight bearing occurs through the forefoot. The normal sequence of development of walking does not include walking on toes and generally proceeds gradually to a heel-toe pattern, with a heel-strike present at 18 months and a heel-to-toe gait achieved by age 3 [30]. Sometimes this process is compromised with the persistence of toe walking during the growth, as in the ASD child. W.J. Barrow et al. [31] confirmed the high incidence of toe walking in ASD and with language disorders and also raised the possibility of a secondary orthopedic deformity that can complicate long-term management of these patients. The largest study in the literature [32] on Patient Record Database (2,221,009 pediatric patients) revealed 8% of ASD children had toe-walking. These study showed that typically developing children have higher rates of toe-walking resolution without treatment than children with ASD, and therefore the latter require more attention from the doctor.

In addition, important correlations between ASD and musculoskeletal disorders have been studied. Ludyga et al. [33] demonstrated ASD children had impairments in executive function and muscle strength compared to healthy peers. Higher muscle strength was independently associated with better executive function but only in ASD patients. The promotion of muscle strength, for example, by regular exercise, could contribute to a reduction of ASD-related executive dysfunction. Stins et al. [34] reviewed motor abnormalities, such as poor timing and coordination of balance could affect children with ASD. Furthermore, other muscular diseases in young children with ASD, such as hypotonia, could be an early marker for higher autistic symptom severity and lower quality of life. Hypotonia had more common motor stereotypes and a later onset of independent walking [35]. ASD patients frequently reported fatigue, sensory alterations, and coordination. Hypermobility was ascribable to muscle hypotonia, probably characterized by excessive tendon elasticity. The clinical presence of hypotonic muscles was correlated with altered proprioception sensorially, which interfered with motor sequence planning. This was a new hypothesis “Connectivome Theory” from Zoccante et al. [36] and explained alterations in the physiological activity of microglia could be implicated in the pathogenesis of ASD.

This report has encouraged us to better investigate this disease and, in our work, in a series of 40 ASD children, only 30 met all inclusion criteria. We selected 22 children in the end. The Toe Walking Tool and the family/patient compliance were important indicators to enroll patients. The work mainly used the orthopedic point of view, without neglecting the whole ASD child.

The literature showed conservative and surgical treatments concerning different modalities. The surgical treatment often is not optimal in ASD patients because of the high recurrence rate and it is reserved for severe cases. Conversely, there are many conservative options such as orthoses. However, several studies showed that the use of orthoses alone is not sufficient to obtain an adequate correction [37]. Herrin K. et al. [15] studied a toe walking patients group treated with orthoses only: they demonstrated high risk of the return of pathological walking after the treatment.

We consequently decided to propose a non-surgical protocol. The “Cast and Go” protocol combined the casts action with orthoses and “broad spectrum” rehabilitation. The literature demonstrated how serial casts allowed the sural muscles to stretch in a physiological way to follow the bone growth of the leg [38,39]. This method reproposed to the sural muscles the same stresses and vector forces to which they are subjected during the child’s growth [40]. Physiologically, bones increase in length thanks to the growth epiphyses, while the muscles follow the growth due to an elongation mechanism. Therefore, the goal was to follow the ASD child until the end of the growth (about 18 years) to avoid a pathological foot. A continuous stretching period in plaster (about 30 days) for the sural muscles simulates the physiological pathway as during the child growth. Following this rationale, the plaster corrected the deformity achieving the neutral position of the ankle. Although our data showed a recurrence rate of the disease after first protocol application from 50 to 66.6%, this did not influence the final result. The deformity correction took place gradually allowing a physiological adaptation of both the soft structures [39] and the perception of the child itself [41]. In fact, all patients completed the protocol reaching 90° of dorsiflexion (100% of the sample).

Regarding the botulinum toxin role, the literature described the non-improvement of casting results [42,43]. Notwithstanding this, it was included in our protocol because we had better results in making the first cast, maybe thanks to the greater “compliance” of sural muscles and because the child, often, did not tolerate the stretch position in plaster for a long time [44].

There were few studies that demonstrated the efficacy of this combination treatment for ASD toe walking; most of these had a small number of cases. However, they demonstrated the effectiveness of the combination of serial casting and orthoses associated with behavioral therapy [41]. The role of family and patient compliance is fundamental: they must work as a team so that the multidisciplinary approach (physiotherapists, psychologists, occupational therapists and orthopedics) increases the effectiveness of the treatment. Unfortunately, two patients 2 (9.09%) were lost to follow-up after first protocol application. They were handed to local social services and placed in a community. This situation made multimodal treatment difficult. These patients were referred to the Psychological Department. An important task for therapists is the recovery of proprioception and sensitivity which are impaired in the child with ASD [8]. From the different approaches used in our department, occupational therapy is one of the most involved. The Hospital Psychology Department provides periodic sessions for the family and for the child during the whole treatment. Our patients were usually treated by a Unit Physiotherapist, at least twice a week throughout the standardized protocol. Family-centered rehabilitation therapies are positively associated with greater participation in family/recreation activities and walking endurance. Parental perception that rehabilitation therapies meet children’s needs was associated with greater participation in family/recreation activities. Structured play, recreational activities and health/well-being were important for self-care and participation when planning rehabilitation therapy. Williams and Haines [45] showed the impact on quality of life of toe walker patients and their family, comparing it with a healthy population, cerebral palsy and attention and hyperactivity disorder (ADHD). They found no significant impact on quality of life of toe walker as compared to cerebral palsy and ADHD populations.

Changes in ASD features were additionally evaluated using GARS-3 in order to compare the whole patient condition before and after treatment. The literature demonstrated unique ASD score has not successfully been reached; on the other hand, GARS-3 was widely used in Western countries [28]. Secondly, there were no studies evaluating changes in ASD children with ITW after orthopedic treatment. The literature tended to focus on the orthopedic or neuropsychological aspect only but not to integrate them together [46]. We demonstrated no statistically significant differences after treatment with our protocol.

Some factors can influence the prognosis of toe walker ASD, such as the age, severity of equinus, the child and family compliance. Age is probably the most important variable in achieving 90 degrees of dorsiflexion. Engström P. et al. [47] showed a trend and prevalence of tiptoe walking from birth to 10 years of age: by the age of 10 years, 79% of the children who have ever been a toe-walker spontaneously develop a typical gait without intervention or contractures of the ankle dorsiflexion; this child should be treated early in childhood. For this reason, in our study, we demonstrated that the younger they are, the better and longer lasting the results. Moreover, we underlined there is no significant difference between male and female children, although toe walking is more frequent in male patients that started with worse dorsiflexion angles. The severity of equinus is a truly important factor and can influence the whole treatment: it is related to the casting number necessary to obtain neutral ankle position. De facto, we demonstrated the baseline angle statistically influenced treatment: the larger the angle, the more plasters were needed to obtain the correction. Group 3 needed our protocol two or three times, but all patients at the final removal of the plaster had reached 90°.

This study has some important limitations. The sample size is limited and not sex homogeneous. Probably an intrinsic bias could be the number of protocols applied in the different groups, even if this same data are statistically significant with the baseline ankle starting angle. Finally, pedobarographic examination could be applied. In fact, the analysis of pressure data using a parametric mapping is higher in resolution and can enhance understanding of foot function development, providing novel insights into plantar pressure changes before and after the treatment [48]. On the other hand, we presented a new treatment protocol, which has proven effective and is shown in the data observed. Secondly, we emphasized the team role (different specialists around child and family). Thirdly, we performed a rigorous method of patient selection with inclusion/exclusion criteria and the use of the “Toe Walking Tool”. Our results should be confirmed by a large sample size prospective randomized controlled study.

## 5. Conclusions

The “Cast and Go” protocol showed one possible conservative treatment in ASD idiopathic toe walking patients. We underline that the larger the baseline ankle angle, the more plasters were needed. Unfortunately, pre-adolescents had more recurrence risk. The botulin toxin, orthoses and rehabilitation play a secondary but fundamental role to manage toe walkers.

We considered interprofessional collaboration between doctor, psychologist, family and therapists to be important as the main element of treatment.

The data observed in this study should be improved by a prospective randomized controlled study.

## Figures and Tables

**Table 1:** Main data of the study.

**Age**	
Mean ± SD	9.05 ± 3.47
Range	4–15
**Gender**	
Male. n (%)	12 (54.5%)
Female. n (%)	10 (45.5%)
**Age subgroup (year)**	
4–7. n (%)	8 (36.4%)
8–11. n (%)	8 (36.4%)
12–15. n (%)	6 (27.3%)
**Baseline Ankle-dorsiflexion angle (°)**	
Mean ± SD	103.18 ± 8.67
Range	90–120
**Casting number as treatment**	
One. n (%)	13 (59.1%)
Two. n (%)	5 (22.7%)
Three. n (%)	4 (18.2%)
**Recurrence**	
Yes. n (%)	12 (54.5%)
No. n (%)	8 (36.4%)
**Lost to follow up. n (%)**	2 (9.09%)
**Test di Kolmogorov–Smirnov**	***p* < 0.05**

Ten patients.

**Table 2:** Correlation between sex and recurrence (tau-b Kendall), comparison between sex and baseline ankle-dorsiflexion angle (°), casting number as treatment (Mann–Whitney U test).

	Males (12 Patients)	Females (10 Patients)	*p*
**Baseline Ankle-dorsiflexion angle (°)** Mean ± SD	107.50 ± 7.54	98 ± 7.14	**<0.01**
**Casting number as treatment** Mean ± SD	1.75 ± 0.75	1.40 ± 0.84	0.17
**Recurrence** (%)	50%	60%	0.28

**Table 3:** Correlation between group and recurrence (tau-b Kendall), comparison between sex and baseline ankle-dorsiflexion angle (°), cast numbers as treatment (Kruskal–Wallis test).

	Group 1 (4–7 y, 8 Patients)	Group 2 (8–11 y, 8 Patients)	Group 3 (12–15 y, 6 Patients)	*p*
**Baseline Ankle-dorsiflexion angle (°)** Mean ± SD	97.50 ± 2.67	107.5 ± 11.65	105.00 ± 5.48	0.08
**Casting number as treatment** Mean ± SD	1 ± 0	2.25 ± 0.89	1.50 ± 0.55	0.10
**Recurrence** (%)	50%	50%	66.6%	0.51

**Table 4:** Correlation between baseline ankle-dorsiflexion angle (°) and casting number as treatment, recurrence (tau-b Kendall).

Baseline Ankle-Dorsiflexion Angle (°)	Casting Number as Treatment	*p*	Recurrence	*p*
100	1	**0.001**	No	0.17
95	1		No	
100	1		Yes	
95	1		Yes	
110	2		No	
110	3		Yes	
120	3		Yes	
90	1		No	
110	2		Yes	
100	1		Yes	

## Data Availability

The data presented in this study are available on request from the corresponding author. The data are not publicly available due to privacy.
